# Tuning the Optical Properties of Electrospun Poly(methyl methacrylate) Nanofibres via Montmorillonite and Magnetite Ratios

**DOI:** 10.3390/polym17030384

**Published:** 2025-01-31

**Authors:** Yao Mawuena Tsekpo, Weronika Smok, Krzysztof Matus, Barbara Hajduk, Adrian Radoń, Paweł Jarka, Tomasz Tanski

**Affiliations:** 1 Department Engineering Materials and Biomaterials, Faculty of Mechanical Engineering, Silesian University of Technology, Konarskiego 18A, 44-100 Gliwice, Poland; weronika.smok@polsl.pl (W.S.); pawel.jarka@polsl.pl (P.J.); tomasz.tanski@polsl.pl (T.T.); 2 Materials Testing Laboratory, Faculty of Mechanical Engineering, Silesian University of Technology, Konarskiego 18A, 44-100 Gliwice, Poland; krzysztof.matus@polsl.pl; 3 Centre of Polymer and Carbon Materials, Polish Academy of Sciences, 34 Marie Curie-Skłodowska Str., 41-819 Zabrze, Poland; bhajduk@cmpw-pan.pl; 4 Łukasiewicz Research Network—Institute of Non-Ferrous Metals, Sowińskiego 5 St., 44-100 Gliwice, Poland; adrian.radon@imn.lukasiewicz.gov.pl

**Keywords:** composite, nanofibres, optical properties, PMMA, montmorillonite, magnetite, electrospinning

## Abstract

Poly(methyl methacrylate) (PMMA) polymer has unlocked new frontiers in the field of nanotechnology and is suitable for a wide range of applications. However, its optical band gap limits its use in optoelectronics. This study aims to ascertain the influence of varying montmorillonite and magnetite ratios on the optical properties of electrospun PMMA nanofibres produced from solution. The nanofibres were characterised using Fourier-transform infrared spectroscopy (FTIR), atomic force microscopy (AFM), scanning electron microscopy (SEM), X-ray diffractometry (XRD), energy-dispersive X-ray spectroscopy (EDX), spectroscopic ellipsometry, and UV-Vis spectroscopy (UV-Vis). XRD analysis revealed the successful incorporation of magnetite and montmorillonite within the PMMA matrix, with diameters ranging from 203 to 328 nm. The incorporation of magnetite and montmorillonite altered the light absorption characteristics of PMMA, resulting in increased absorption in the ultraviolet and visible light regions compared to pristine PMMA and a reduction in the optical band gap from 4.9 eV to 2.5 eV. These findings suggest that PMMA is a suitable host matrix for montmorillonite and magnetite. The observed properties also indicate the suitability of the produced materials for optoelectronic applications, including chemical sensors and protective UV coatings.

## 1. Introduction

A diverse array of polymers is commonly utilised for various applications in optoelectronics [1,2,3]. Among these is poly(methyl methacrylate) (PMMA), a versatile polymer renowned for its exceptional optical properties, rendering it a prevalent choice in numerous applications, including optoelectronics and photonics. Initially discovered in the early 1930s by British chemists, Rowland Hill and John Crawford, followed by its first application by a German chemist, Otto Rohm, in 1934, PMMA is an optically transparent thermoplastic [4]. It is extensively employed as a substitute for inorganic glass due to its high impact strength, lightweight, shatter resistance, and favourable processing conditions [5,6,7]. However, its optical transparency and electrical insulation properties are what primarily make it a suitable candidate for optoelectronic applications. For these reasons, PMMA is being explored in various fields such as optics, telecommunications, and the production of general-purpose products.

A variety of processing techniques are employed to optimise the optical properties of PMMA. Commonly used methods that yield desirable outcomes include spin coating [8], solution casting [9], electrospinning [10], and hot injection and ligand exchange [11]. Among these techniques, electrospinning offers distinct advantages for producing PMMA with superior optical properties. Notably, electrospinning provides a high surface area-to-volume ratio, which is crucial for applications requiring high optical clarity and enhanced light interaction, such as optical devices and sensors [12]. Additionally, the ability to achieve controlled morphology allows for the creation of nanofibres with diameters ranging from tens to hundreds of nanometers while maintaining uniform alignment; this maximises the quantum effect [13]. Because of these advantages, scientists are exploring electrospinning as a viable manufacturing method for achieving PMMA with superior optical properties.

Electrospinning has been used to create novel materials for several applications, such as environmental protection and cleanup. Numerous sectors, including universal membranes, scaffolding and wound treatment for biomedical devices, composite reinforcement, and high-surface-area fabrics for sensors and protective gear, have increased their use of electrospun nanofibrous mats [14,15]. The preparation of fibre is significantly influenced by the properties of the polymer, such as viscosity, concentration, surface tension, conductivity, and molecular weight [16]. In the case of electrospinning equipment, the tip-to-collector distance, voltage intensity, flow rate, collecting roller speed, temperature, and humidity all play a critical role in the successful electrospinning process [17]. An electric field is generally created using an electrical charge to draw the polymer from the syringe to the collector during electrospinning. The tip of Taylor’s cone, which would develop due to the applied electric force, would shoot a charged fluid jet [18]. The diameter of the jet decreases as it travels through the air due to solvent evaporation, jet stretching, and the extension rate [19].

The applications of PMMA in the literature can be observed in reviews reported in recent years [5,20,21,22,23,24,25]. A consistent trend identified in these reviews is the modification of PMMA, typically achieved by incorporating nanoparticles and nanostructures within PMMA’s matrix. This incorporation leads to composites with enhanced properties such as expanded surface area, novel architectures, surface selectivity, surface energy, light absorption, and a high strength-to-weight ratio. Common examples of nanoparticles and nanostructures used in the modification of PMMA include TiO_2_ [26,27], ZnO [20,28,29], Ag [30,31], Au [32,33], Nb_2_O_5_ [34], Al_2_O_3_ [35,36], carbon nanotubes [37,38,39], carbon nitride [40], and nanoclays [41,42,43]. Mixtures of these nanoparticles and nanostructures have also been reported in the literature, demonstrating significant improvements in the properties of PMMA. For instance, Das et al. explored the influence of TiO_2_ and cloisite on PMMA and reported improved dielectric properties [44]. Additionally, the incorporation of nanoparticles and nanostructures can alter the optical properties of PMMA, as evidenced by the work of Mohammed et al., who observed a decrease in the optical bandgap of PMMA with increasing concentrations of ZnO [20]. These examples illustrate that PMMA composites with nanoparticles or nanostructures have many applications.

Magnetite is a naturally occurring iron oxide with a chemical formula of Fe_3_O_4_. Due to its unique properties, it is readily available and easily utilised, particularly in the nanoscale regime; these properties include superparamagnetism at ambient temperature, high reactivity from its large active surface area, and high stability in aqueous solutions [45]. These characteristics render magnetite suitable for applications in the biomedical field for drug delivery systems and contrast agents during MRI [46], as well as in environmental applications for water purification and remediation, owing to its ability to adsorb contaminants [47]. A property of magnetite that researchers are currently investigating is its optical characteristics, as demonstrated in the work of Akhtar et al., who examined the optical properties of green-synthesised magnetite nanoparticles and evaluated the degradation of methylene blue dye via photocatalysis [48].

Montmorillonite (MMT), with the chemical formula (Na, Ca)_0.3_(Al, Mg)_2_Si_4_O_10_(OH)_2_·n(H_2_O), is a highly malleable phyllosilicate group of minerals, and a primary constituent of montmorillonite has been the subject of extensive research for decades. Its Cation Exchange Capacity (CEC), adsorption characteristics, biocompatibility, and antimicrobial activity when combined with metal or metal oxide nanoparticles are properties that render it suitable for a wide range of applications. These include biomedical applications as a material component in drug delivery systems, tissue engineering, and as antimicrobial agents. Additionally, in environmental applications, it is effective in adsorbing pollutants from water and soil, making it valuable in remediation technologies. In industrial applications, it is employed as an additive in polymers to enhance mechanical properties or as a support for catalysts in chemical reactions. Ammar and Fakhfakh reported the use of montmorillonite in the reduction in the optical band gap of polypropylene, providing the scientific community with insight into new opportunities to apply montmorillonite [49].

However, over the years, the literature demonstrates instances where magnetite and montmorillonite have separately been investigated in conjunction with PMMA as composites for various applications, yielding noteworthy properties [42,50,51,52,53,54]. Similarly, in the context of composite nanofibres, magnetite and montmorillonite have been examined separately by researchers. Notable among the published works is Zhang et al. [55] who studied the dielectric properties of electrospun PMMA/magnetite composites and reported an increased dielectric permittivity with the inclusion of magnetite. The membranes exhibit superparamagnetic properties and a decreasing dielectric permittivity with increasing Fe_3_O_4_ nanoparticle content. These findings suggest potential applications in reducing propagation delay and cross-talk noise in ultra-large-scale integrated circuits. Additionally, Wang et al. [56] found that cationic surfactants with a methacryl-tethering group significantly enhance the exfoliation of montmorillonite clay in PMMA nanocomposites. This enhances the electrospinnability of polymer/clay dispersions, resulting in smaller diameter fibres. The total solids concentration influenced the diameter of electrospun fibres, while clay content had minimal impact. This suggests a novel method for producing finer fibres for small-diameter applications.

Building upon previous work, this paper presents the successful preparation of PMMA/montmorillonite/magnetite composite nanofibres using the electrospinning technique. The composite nanofibres were thoroughly characterised to understand their optoelectronic properties and morphology.

The innovative aspect of this research lies in the use of nano-reinforcement to enhance the optoelectronic characteristics of electrospun mats. This study is one of the first to investigate the synergistic effects of both nanofillers on the morphological and optical properties of PMMA nanofibres. The findings provide new insights into how montmorillonite and magnetite nanoparticles can modify and improve the optoelectronic performance of electrospun nanofibres, highlighting their potential applications in advanced materials and optoelectronic devices.

## 2. Materials and Methods

### 2.1. Materials

Without further purification, montmorillonite clay powder (<50 nm, Sigma-Aldrich, Dorset, UK, item number 682659) was used as received. Green synthesised was also utilised to make iron oxide (Fe_3_O_4_) nanoparticles prepared according to the method laid out in Tsekpo et al. [57]. Analytical grade ferrous chloride FeCl_2_.4H_2_O (Molar mass = 198.81 g/mol, ≥95% purity), ferric chloride FeCl_3_.6H_2_O (Molar mass = 270.33 g/mol, ≥95% purity), and sodium hydroxide (NaOH, molar mass = 40 g/mol, ≥95% purity) were obtained from Sigma-Aldrich. Maize leaf extracts were also employed to guarantee that the synthesis was environmentally friendly. They were made in compliance with the guidelines provided by Asimeng et al. [58]. Poly(methyl methacrylate) (PMMA, 99%, Mw = 9,600,000) was obtained from Sigma-Aldrich as well as N, N-Dimethylformamide (DMF, 99.8% purity).

**Figure 1:**
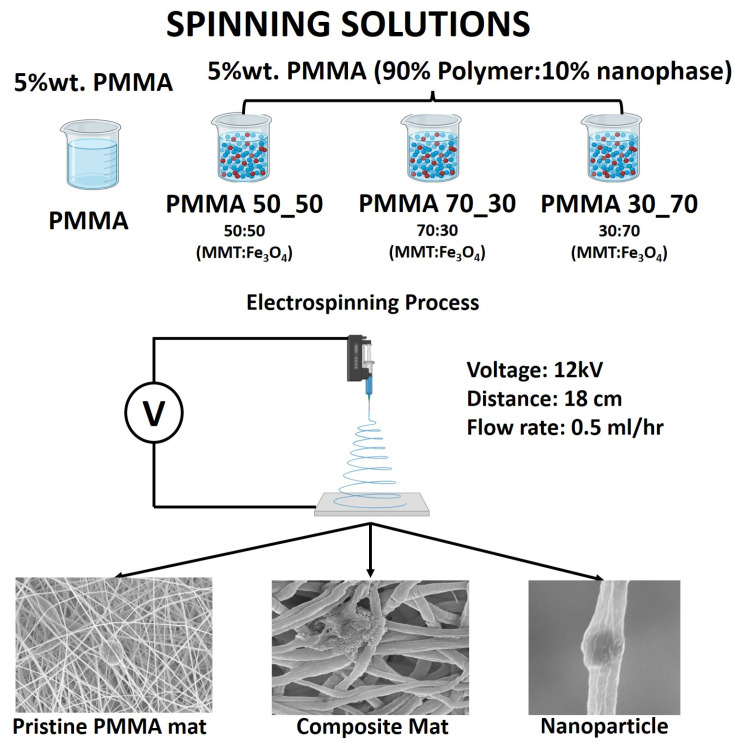
Schematic representation of the methodology.

The final products were the solutions of PMMA/DMF/montmorillonite/Fe_3_O_4_ at a concentration of 5% by weight of PMMA, with a 10% concentration of nanoparticle by weight mixed in 3 different ratios of montmorillonite: magnetite, 50:50, 70:30, and 30:70 (Figure 1, labels used in this figure will be constantly referenced throughout this paper) to ascertain the synergistic effect of both nanofillers on the morphology and optical properties of electrospun PMMA. In order to break the agglomerates of the nanoparticles, the measured amount of reinforcing phase was added to DMF; so, prepared solutions were subjected to sonification for 15 min. Directly after the sonification process, the measuring amount of polymer was added to the sonicated solution and subjected to mixing using a magnetic mixer for 24 h at room temperature. Immediately after mixing, the solution was placed in a pump device, which was a sterile syringe. The polymer and composite nanofibres were obtained using the electrospinning method from the solution using the device FLOW—Nanotechnology Solutions Electrospinner 2.2.0-500 (Yflow, Malaga, Spain). The process parameters are voltage and distance between electrodes, a flow rate of 12 kV, 18 cm and 0.5 mL/h, respectively.

### 2.2. Characterisation

A scanning electron microscope (SEM, SUPRA 35 by ZEISS, Oberkochen, Germany) with the EDAX Trident XM4 series X-ray spectrometer (EDX) was used to observe the morphology of the nanoparticles and the prepared fibrous mat. EDX on the SEM was only used in the examination of the prepared fibrous mats. Samples were sputtered with gold on their surface before their observation under the microscope.

Transmission electron microscopy was performed using Titan G2 80- 300 kV (FEI Company, Hillsboro, OR, USA) equipped with the field emission gun (FEG), beam corrector (Cs-corrector), HAADF detector, and EDS (energy-dispersive X-ray spectroscopy) spectrometer used in the investigation of the nanoparticles.

The topography and surface roughness of the fibrous mat were analysed using atomic force microscopy (XE-100 by Park Systems, Suwon, Republic of Korea) controlled with XEI Software (version 5.2.4). This software enables the processing of images and analysis of the surface roughness parameters. The microscopic measurements were taken in non-contact mode.

The state of the chemical bonds present in the nanoparticles and fibrous mats was analysed using Fourier-transform infrared spectroscopy (Nicolet 6700 from Thermo Scientific, Waltham, MA, USA) in the Attenuated total reflectance (ATR) mode.

The presence of montmorillonite and magnetite nanoparticles in synthesised samples was confirmed using X-ray diffraction (XRD). For this purpose, the XRD patterns of composite samples and pure components (PMMA, montmorillonite clay, and Fe_3_O_4_ NPs) were measured using a Rigaku MiniFlex 600 diffractometer (Rigaku Corporation, Tokyo, Japan) equipped with copper tube Cu Kα (λ = 0.15406 nm) as a radiation source (tube voltage 40 kV, current 15 mA). The phase composition was analysed using the dedicated Rigaku PDXL 2 v.2.9.2.0 software suite.

Thermo-UV/VIS Scientific’s Evolution 220 spectrophotometer (Madison, WI, USA) was used to measure the absorbance spectra versus wavelength to determine the optical characteristics of the nanoparticles, fibre polymers, and composite mats. The optical absorbance of nanoparticles was determined by dispersing 1 mg of the nanoparticle in 10 mL ethanol and applying a wavelength of light ranging from 190 to 1100 nm perpendicularly to the sample. The optical absorbance of the nanofibrous mat was determined by the method laid out in [59,60,61]. The spectral data obtained from the UV/VIS and Tauc plot given by the equation below, the optical band gap of the nanoparticles, and fibre polymer and composite mats were determined in the fashion as reported by [62,63,64].

(1) $$\alpha h\upsilon =A{\left(h\upsilon -E_{g}\right)}^{\rho }$$

where *α*—absorption coefficient, *h*—Planck constant, *υ*—electromagnetic radiation frequency, *A*—constant, *hν*—photon energy, *E_g_*—energy band gap, and *ρ*—coefficient that characterises the transition in the K-space.

The ellipsometric measurements were carried out using the spectroscopic ellipsometer SENTECH SE850E (Berlin, Germany), working in the 240–2500 nm range, operated with Spectra Ray version 3 software. The measurements were taken in two modes—the transmission and variable angle mode. The transmission spectra were taken on films deposited onto quartz substrates and the ellipsometric measurements—Ψ and Δ—were taken in the 40–70° angle range, measuring every 10°.

## 3. Results and Discussion

### 3.1. Morphology

**Figure 2:**
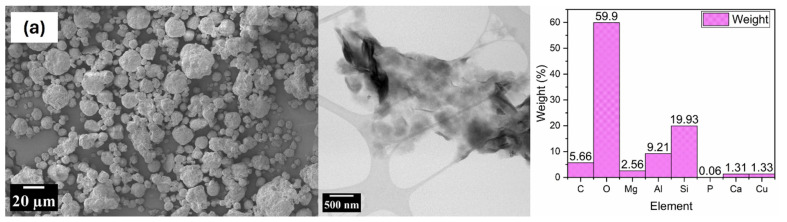

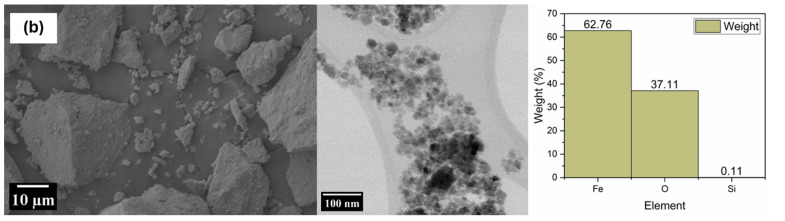
SEM and TEM with EDX elemental analysis of MMT (**a**) and magnetite (**b**).

Figure 2 shows the SEM and TEM accompanied with EDS of MMT and magnetite nanoparticles used in the preparation. Although both nanoparticles are observed to form agglomerates under the SEM, the magnetite nanoparticles are noted to be angular in shape whereas that of MMT is spherical.

Further examination under a TEM revealed the nanostructure of MMT, revealing platelets with irregular morphologies characteristic of distinct clay particles. These platelets, varying in size and shape, are emblematic of diverse clay minerals [65]. Notably, some platelets are observed to overlap or stack, a hallmark trait of specific clay minerals. This may enhance the surface area accessible for adsorption, making montmorillonite beneficial in applications such as wastewater treatment [66]. Interspersed darker regions suggest the presence of voids or pores, which are pivotal in influencing attributes like moisture retention in clays [67]. Additionally, particle aggregation is evident, potentially driven by electrostatic interactions or the intrinsic tendencies of certain clay minerals. The presence of voids coupled with particle aggregation within the material affect the packing and porosity of MMT [68]. The EDS analysis of nanostructure MMT revealed the presence of aluminium (Al), calcium (Ca), magnesium (Mg), silicon (Si), and sodium (Na), all of which are characteristic elements present in MMT.

The TEM images of the nanostructure of Fe₃O_4_ particles offer invaluable insights into their morphological and dimensional attributes. From a preliminary assessment, the particles distinctly exhibit a polydispersed distribution, encompassing a range of sizes. This is typical in many nanoparticle systems and can affect attributes like reactivity and stability [69]. While their core structure largely aligns with spherical morphology, subtle variations in shape can be discerned upon closer examination. This could be due to factors such as the synthesis method or conditions [70]. A notable observation is the marked agglomeration of several nanoparticles, forming conspicuous clusters. This tendency for aggregation can be ascribed to magnetic interactions inherent to magnetite or potentially van der Waals forces. The agglomeration impacts the effective surface area of the nanoparticles for reactivity [69]. The presence of discernible inter-particle spaces could signify the role of stabilising agents, which function to deter extensive coalescence and maintain nanoparticle dispersion. The EDS analysis of these particles revealed the presence of iron (Fe) and oxygen (O), which are evidence of the presence of magnetite.

**Figure 3:**
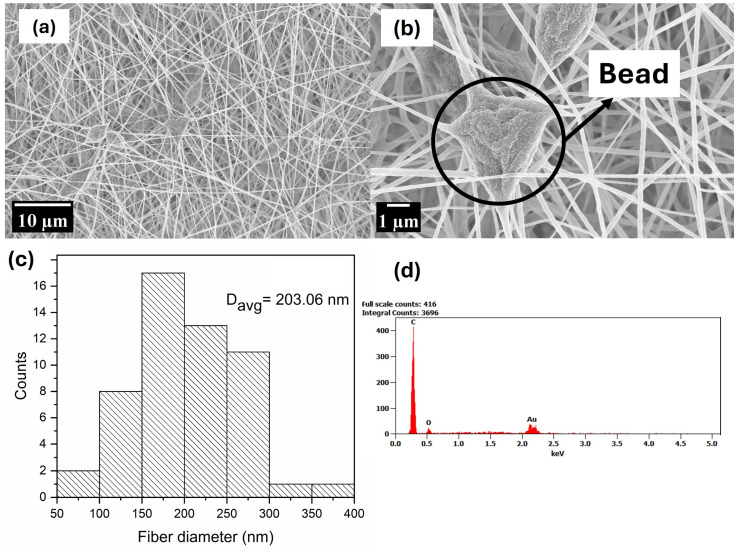
Image (**a**,**b**), (**c**) fibre distribution, and (**d**) EDX analysis of PMMA.

**Figure 4:**
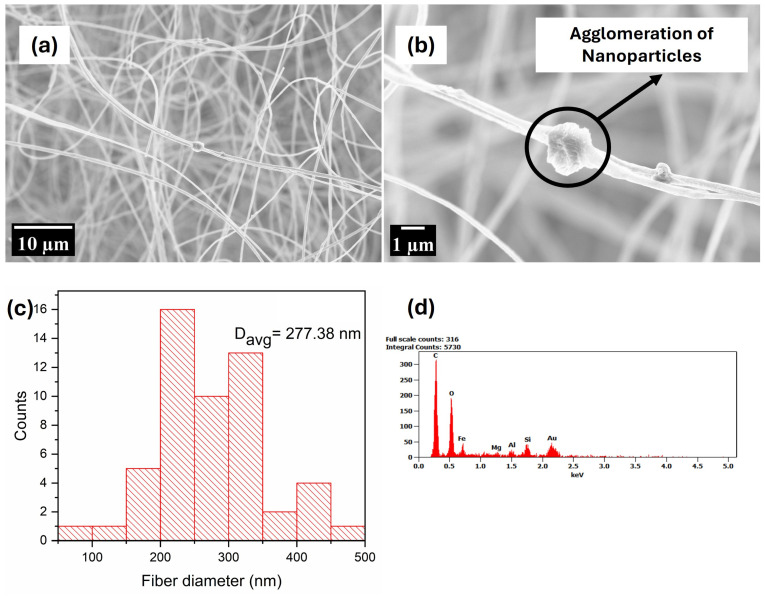
Image (**a**,**b**), (**c**) fibre distribution, and (**d**) EDX analysis of PMMA 50_50.

**Figure 5:**
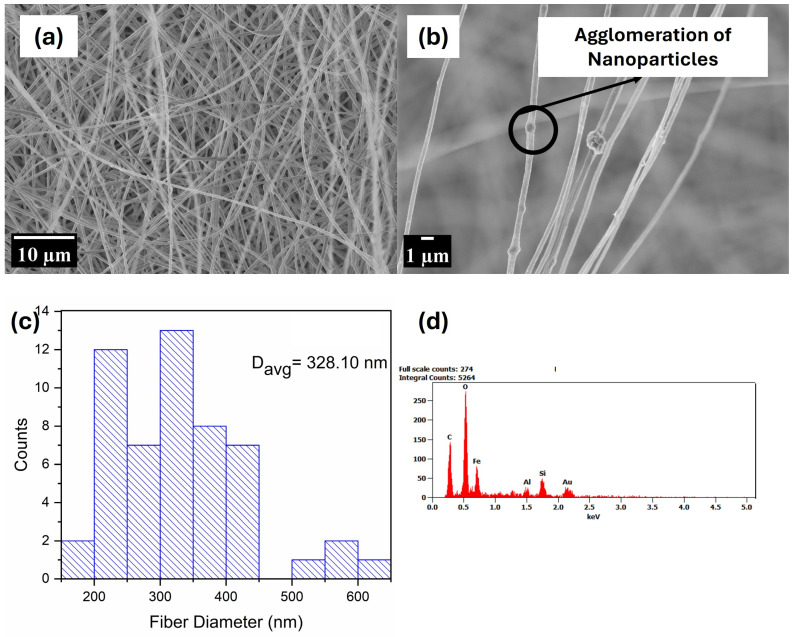
Image (**a**,**b**), (**c**) fibre distribution, and (**d**) EDX analysis of PMMA 70_30.

**Figure 6:**
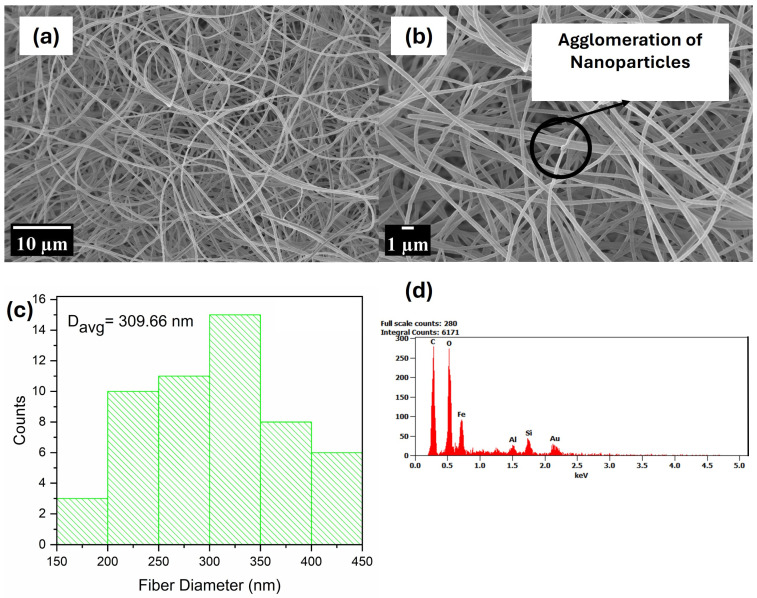
Image (**a**,**b**), (**c**) fibre distribution, and (**d**) EDX analysis of PMMA 30_70.

Figure 3, Figure 4, Figure 5 and Figure 6 display SEM images of the prepared PMMA fibres, the composite fibres made from solutions of this polymer with various nanophase ratios, and their elemental composition as characterised via EDX. The average diameters of the nanofibres were estimated using ImageJ software (version 1.54m). For each micrograph, the diameters of 53 fibres were measured, and their average fibre diameter is shown in plot distribution. It can be observed that the fibres produced from pristine PMMA were achieved in the nano regime; although smooth fibres can be visibly seen, the presence of beads is also noticed. The presence of beads can be attributed to the low concentration of PMMA used in the electrospinning process, which reduces the viscosity of the spinning solution, hence the low entanglement of the polymer chains in the solution [71]. The low entanglement of polymer chains in solutions creates a capillary instability at the electrospinning jet [72]. This leads to the formation of beads as a result of electrostatic scattering, which creates an electrospraying effect. This phenomenon is consistent with reports by [71,72,73] who observed the presence of beads in electrospun PMMA as a function of low concentrations. The EDX analysis showed the presence of carbon (C) and O, which are elements that are characteristic of PMMA.

The micrographs in Figure 4, Figure 5 and Figure 6 point to the increased fibre diameter upon the introduction of nanophase as compared to pristine PMMA. Comparing the composite fibres to the pristine fibre, it can be observed that there are fewer beads present and the fibres are neat and uniformly shaped. Also, it can be observed that the nanophase forms agglomerate within the fibres produced, although there are portions within the fibrous mat exhibiting uniformity between the polymer and nanophase. However, in comparison to the pristine PMMA fibres, the composite fibres are noticed to be more rough, which can be attributed to the difference in particle morphology of the nanophase. The increase in diameter of the fibres can be attributed to the increased viscosity of the doped solution caused by the nanophase altering the composition of the spinning solution [74]. This alteration will dilute the concentration of the spinnable polymer in the spinning solution whilst increasing the solid concentration within the solution and, hence, the viscoelastic properties of the spinning solution [75]. This observation in composite fibre was also seen by Wutticharoenmongkol and team [74]; they observed the increase in the fibre diameters of polycaprolactone (PCL) upon the addition of nano-hydroxyapatite (HA)/calcium carbonate (CaCO_3_). Similarly, works by [75,76,77] also confirm this observation with concurring explanations. The EDX analysis showed the presence of Fe, O, Ca, Si, C, Na, and Al; elements that point to the successful production of a composite fibrous mat.

**Figure 7:**
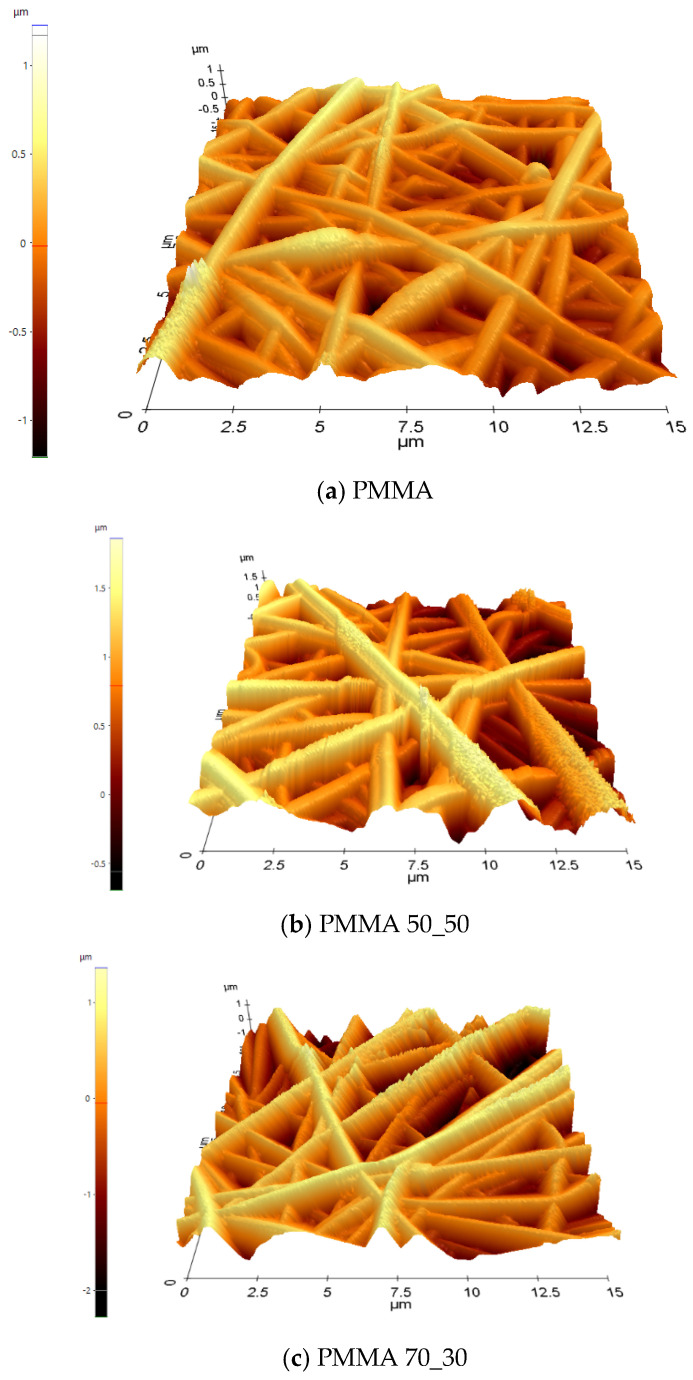

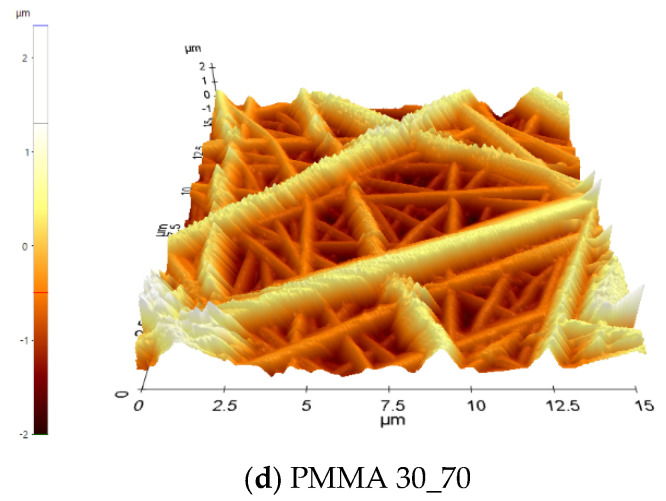
Three-dimensional AFM images of samples (**a**–**d**).

AFM is regarded as a widely utilised method for characterising nanofibrous materials since it provides details on the topography, shape, and distribution of fibres on the sample surfaces. As a result, atomic force microscopy in non-contact mode was used further to examine the surface morphology of the nanofibrous mats. In this study, an AFM scan range (15 μm × 15 μm) was used to examine the surface roughness of nanofibrous mats, as shown in Figure 7 and Table 1. The topography of the nanofibres seems to be a rather ordered overlap of fibres; also, the roughness coefficient is observed to be high for samples. The minimum value readings for all samples were negative, indicative of the porous nature of the mats leading the probe below the surface of examination.

The root mean square roughness (R_q_) is a reflection of the valleys and peaks present on the surface of fibres and can be correlated to the surface area of the material in some instances. The observed porosity and roughness, particularly in the composite samples, make the nanofibrous mats suitable for applications related to interactions with other materials. Such applications include chemical sensors and coating where the roughness provides an active surface area for material interaction and chemical reactions like extracting a certain component of a liquid or gaseous substance.

The increase in roughness with the addition of nanoparticles was observed by Bulbul et al. when they employed loaded poly (lactic acid) (PLA)/polyvinylpyrrolidone (PVP)/TCH-multiwall carbon nanotube (MWCNT) composite fibrous mats to study the drug release of tetracycline hydrochloride (TCH) [78]. HMTShirazi and team prepared an electrospun fibrous mat from amine group grafted halloysite nanotubes and a polymer blend of chitosan and poly (vinyl alcohol) (PVA) [79]. In their work, it was also observed that surface roughness samples increase upon the addition of halloysite nanotubes to the polymer matrix. This provides an increased surface area for the adsorption of Pb and Cd ions from solution.

### 3.2. XRD

**Figure 8:**
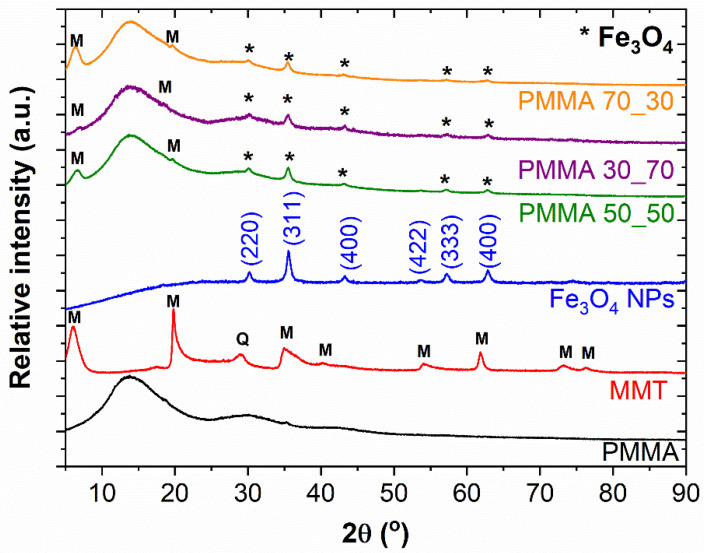
XRD patterns.

The XRD analysis was conducted to evaluate the composition, morphological behaviour, and crystallinity of the PMMA/montmorillonite/magnetite composite nanofibres. The XRD patterns (Figure 8) revealed distinct diffraction peaks corresponding to the crystalline phases present in the composites.

The XRD patterns indicated the successful incorporation of magnetite (Fe_3_O_4_) and montmorillonite within the PMMA matrix. The XRD patterns of Fe_3_O_4_ NPs confirm the one-phase composition of the sample (Fe_3_O_4_; COD card number: 9005839) without any visible impurities related to the formation of other iron oxides, such as hematite. The diffractogram of pristine Fe_3_O_4_ NPs shows characteristic planes (220), (311), (400), (422), (333), and (440), corresponding to the characteristic 2θ peaks of values of 30.1°, 35.5°, 43.1°, 53.4°, 57.0°, and 62.6°, indicating a cubic crystal system belonging to Fd3m (227) space group. This finding is similar to observations made by Yasmine et al. who reinforced epoxy resin with magnetite nanoparticles [80]. In comparison, the montmorillonite clay is characterised by the presence of two phases—montmorillonite with a chemical formula of (Na, Ca)_0.3_(Al, Mg)_2_Si_4_O_10_ (JCPDS card number: 00-003-0015) and quartz (SiO_2_; JCPDS card number: 01-070-3317) marked on XRD patterns as M and Q, respectively. The montmorillonite clay showed peaks at 2θ values of 5.8°, 19.8°, and 26.6°, corresponding to the (001), (110), and (020) planes, respectively. Additionally, the presence of quartz was identified with peaks at 2θ values of 20.9°, 26.6°, and 50.1°, confirming its presence in the composite. The findings are consistent with the work of Saeed and group who synthesised an adsorbent material from raw MMT for dye removal [81]. The matrix, PMMA, was nearly purely amorphous. The presence of the montmorillonite and Fe_3_O_4_ NPs was confirmed for all composite samples. Moreover, the intensity of the diffraction peak at 2θ around 6° follows the changes in the MMT: magnetite ratio, i.e., it is the highest for the sample marked as PMMA 70_30 and the lowest for PMMA 30_70.

The incorporation of magnetite and montmorillonite nanoparticles significantly influenced the morphological behaviour of the PMMA matrix. The XRD patterns showed broadening of the diffraction peaks, indicating a reduction in crystallite size and an increase in amorphous content. The presence of magnetite nanoparticles contributed to the formation of a more ordered structure, while montmorillonite clay introduced a degree of disorder due to its layered silicate structure.

The crystallinity of the PMMA/montmorillonite/magnetite composites was assessed by analysing the intensity and sharpness of the diffraction peaks. The magnetite nanoparticles enhanced the overall crystallinity of the composites, as evidenced by the sharp and intense peaks in the XRD patterns. In contrast, the montmorillonite clay introduced a degree of amorphousness, as indicated by the broadening and reduction in peak intensity. The interplay between the crystalline magnetite and the amorphous montmorillonite resulted in a composite with a unique crystalline–amorphous hybrid structure.

These observations are consistent with recent studies on polymer nanocomposites. For instance, Zhang and O’Connor reported the synthesis and characterisation of PMMA-coated magnetite nanocomposites, where XRD analysis confirmed the successful incorporation of magnetite nanoparticles within the PMMA matrix, leading to enhanced crystallinity [82]. Additionally, a study on montmorillonite nanoclay content in low-density polyethylene composites demonstrated that the inclusion of montmorillonite influenced the crystallinity and barrier properties of the polymer matrix, as evidenced by changes in the XRD patterns [83].

The XRD analysis provided insights into the structural and compositional properties of the PMMA/montmorillonite/magnetite composites. The successful incorporation of magnetite and montmorillonite within the PMMA matrix has significant implications for the material’s properties. The enhanced crystallinity due to magnetite nanoparticles can improve the mechanical strength and thermal stability of the composites. The presence of montmorillonite clay, with its layered silicate structure, can enhance the barrier properties and provide additional reinforcement. The unique crystalline–amorphous hybrid structure of the composites can lead to improved optical, mechanical, and thermal properties, making them suitable for a wide range of applications, including advanced nanocomposites and functional materials.

### 3.3. FTIR

**Figure 9:**
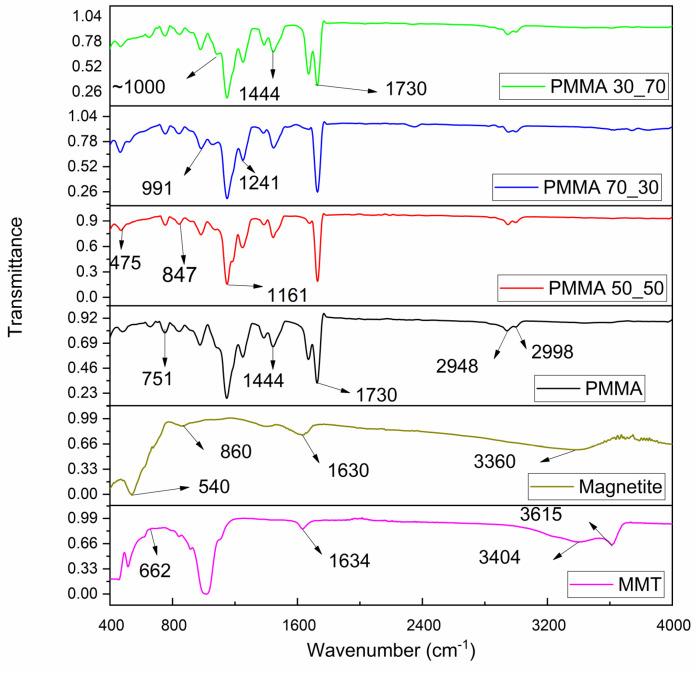
FTIR spectrum of samples.

The absorption bands observed (Figure 9) from the analysis of MMT include 3615, 3404, 1634, 976, and 662 cm^−1^. O–H stretching caused the band at 3615 cm^−1^, while interlayer and intralayer H-bonded O–H stretching caused the broad band at 3404 cm^−1^, which points to the presence of moisture as confirmed in the TEM micrograph. Similar observations were in works by [84,85,86,87] who also attributed this peak to moisture trapped between the layers. At 1634 cm^−1^, a stretching bond is observed with a resemblance to a peak reported by Annan et al. [85] and Tsekpo et al. [57]; this band is characteristic of a siloxane group (-Si-O-Si-), evidence of octahedral and tetrahedral layers bonded by a hydroxyl connection. This hydroxyl connection is observed as a band at wavenumber 1634 cm^−1^, which is attributed to an angular H–O–H bond of water within the interlayer silicate matrix [88]. The bands at 976 cm^−1^ and 662 cm^−1^, respectively, are caused by Al-O and Si-O [89,90,91]. The presence of the above-mentioned bands and the corresponding functional groups, which are supported by the literature and the elemental analysis from the EDS analysis prior, all prove as evidence to support the presence of MMT.

The characterisation of Fe_3_O_4_ particles revealed the presence of bands at 3360, 1630, 1400, 860, and 540 cm^−1^. The broad band at 3360 cm^−1^ is O–H stretching vibration, corresponding to a phenol compound from the maize extracts; this result is in agreement with Mahdavi et al., who observed a peak around 3355 cm^−1^ [92]. The peaks observed at 1630 and 1400 cm^−1^ are consistent with peaks representing proteins and amine, as reported by Naseem et al. [93]. The presence of the phenol, protein, and amine peaks confirms the successful synthesis of Fe_3_O_4_ nanoparticles with bioactive elements. The peak at 540 cm^−1^ verified the presence of iron nanoparticles with a peak analogous to a stretching Fe–O bond vibration. This Fe–O bond stretching vibration was also noted by Wang et al. roughly at 540 cm^−1^ [94]. The presence of the Fe–O bond, amines, and phenols confirms the synthesis of the iron oxide nanoparticles from bioactive ingredients and is in agreement with elemental analysis of the particles via EDS.

The FTIR studies performed on the nanofibres obtained reveal bands present at 2998, 2948, 1730, 1444, 1241, 1161, 991, 847, and 751 cm^−1^ for all samples. The bands at wavenumbers 2998 and 2948 cm^−1^ correspond to the -CH sp^3^ bond in the -CH_3_ and -CH_2_ functional groups [95]. The C=O ester bond is evident at the wavenumber 1730 cm^−1^, evidence of the acrylate carboxyl group [96]. The bands observed at wavenumbers 991, 847, and 751 cm^−1^ are because of a bending -CH bond resulting from α-methyl group vibrations, which are characteristic of PMMA [97]. The band at wavenumber 1444 cm^−1^ can be assigned to the -CH bond from the methyl group, whereas bands observed at 1241 and 1161 cm^−1^ are assigned to C–O–C stretching vibrations [98]. The above-mentioned bands and the corresponding functional groups are indicative of the presence of PMMA. In the composite samples, it is observed that all bands from PMMA are present and visible; however, acute distinct bands were observed at wavenumbers ~1000 and 475 cm^−1^, which can be assigned to the metal hydroxyl group, i.e., Al-OH and Mg-OH, present as a result of MMT and a Fe–O bond contributed by the nano-magnetite [99,100,101]. The presence of these bands are evidence that composite fibres were achieved and confirms the elemental composition obtained from the EDX analysis.

### 3.4. Optical Properties

**Figure 10:**
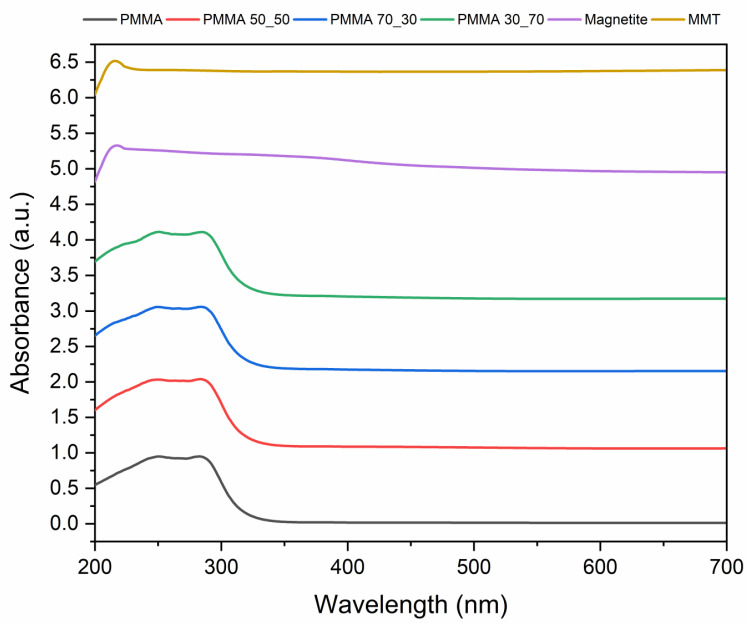
UV-Vis absorption spectra.

Utilising UV-Vis spectroscopy, studies of the resulting mats’ optical characteristics (Figure 10 and Figure S1) were conducted by applying electromagnetic radiation between 200 nm and 1000 nm. PMMA has two absorption bands in its UV-Vis absorption spectrum: one at about 220 nm and the other at about 295 nm. The strong band observed at 220 nm can be attributed to the carbonyl group’s (C=O) π → π* transition. The weaker band at about 295 nm is caused by the carbonyl group’s n → π* transition. The sharp edge absorption is observed in the region of 330 nm, which is indicative of the absorption of ultraviolet electromagnetic radiation. The two absorption bands show that when PMMA absorbs light, two different electronic transitions could take place. The first transition involves an electron moving from a π bond to a π* antibonding orbital, or π → π*. An electron moves from a non-bonding orbital to a pi* antibonding orbital in the second transition, known as an n → π* transition. This finding is consistent with the findings of [102,103,104,105]. The plot reveals that PMMA absorbs substantially at wavelengths less than 250 nm but abruptly loses absorbance at wavelengths greater than 290 nm. This is due to the fact that PMMA allows for two electronic transitions, both of which occur in the ultraviolet (UV) part of the electromagnetic spectrum. This implies that PMMA is transparent within the visible spectrum of electromagnetic radiation and absorbs no light.

Moreover, it was observed that the magnetite nanoparticles prepared via the precipitation technique absorbed electromagnetic radiation in the visible region with a sharp absorption edge at approximately 528 nm and a maximum absorption at 208 nm. This finding demonstrates the strong collaboration between transition metals and plant biomaterials containing hydroxyl groups [106]. The optical band gap was estimated to be 2.2 eV and is consistent with the work of Saad and team [107].

MMT showed absorption peaks at 216 nm, 256 nm, and 350 nm, which are within the middle and near UV regions of the electromagnetic spectrum. The peak at 216 nm is evidence of sites for exchanges between calcium and magnesium valence in the lattice. This absorption peak shows the oxo-to-magnesium charge transfer [108,109]. At 256 nm, the peak corresponds to the π → π* transition Si=O bond in the structure of MMT [108,110,111]. The estimated optical band gap for MMT is 2.5 eV, which is lower than the value reported by Salmanvandi et al. [112], making it a desirable photocatalytic material.

The addition of the nanophase at a concentration of 10% by weight caused a shift to the sharp edge absorption from the ultraviolet region of electromagnetic radiation to the visible light region of electromagnetic radiation observed in the composite samples; however, the intensity of electromagnetic radiation was noticed to decrease upon the inclusion of the nanophase. The presence of the nanophase caused a shift in the sharp absorption edge from the ultraviolet regime of the electromagnetic spectrum to the visible light spectrum. Also, there is a significant reduction in the intensity between the strong and weak absorption bands of PMMA in PMMA 50_50 and PMMA 30_70, whereas that of PMMA 70_30 is only slightly. This occurrence can be attributed to the interaction of the Fe_3_O_4_ and MMT nanoparticles and their interaction with the carbonyl group through electrostatic or Van der Waal’s interactions [113,114,115]. However, from the plots (SI. 1), it is observed that Fe_3_O_4_ is the main contributor to this effect, as shown in the plot of PMMA 70_30. A reduction in the magnetite concentration in the nanophase led to a marginal reduction in the band gap as compared to PMMA 50_50 and PMMA 30_70. The bulk excitation of the nanophase, particularly Fe_3_O_4_ nanoparticles present in the composite fibres, is caused by the shift in the sharp absorption edge and the reduction intensity between the strong and weak absorptions of PMMA [72].

Using the spectra data obtained from the UV-Vis spectroscopy, the band gap of the prepared mats was determined according to the methodology in [62,64]. The *E_g_* obtained for the pristine PMMA nanofibrous mats was approximately 4.9 eV. The outcomes are comparable to those of Thirumala et al. [116]. The resulting PMMA fibres, which formed a thin fibrous polymer mat, were in the nano regime, which is likely what caused the band gap value to shift in the direction of lower energy waves. The magnetite nanoparticles record a band gap of 2.2 eV, which agrees with the works of Ananthi et al. and Saad et al. [106,107]. Using magnetite and MMT nanoparticles in the spinning solution, the composite nanofibrous mats produced are observed to have a lower band gap than pristine PMMA. The lower band gap can be attributed to the enhanced absorption of electromagnetic radiation provided by the introduction of the magnetite phase. This is evident in PMMA 30_70, which is observed to have the lowest band gap and highest concentration of the magnetite phase. It is clear that the obtained composite materials are effective absorbers of ultraviolet radiation while maintaining a precise, unchanged range with respect to the electromagnetic waves absorbed by the matrix mat. Although a decrease in the degree of radiation absorption for a thin composite fibrous mat was observed, it was accompanied by a shift in the sharp absorption edge falling within the visible spectrum of electromagnetic radiation upon the increase in the nanophase from 0 to 10%. This implies the composite nanofibrous mat absorbs light in both the visible and ultraviolet regimes of the electromagnetic spectrum, making it a suitable photocatalyst.

The findings observed are consistent with the known scientific literature. Sengwa and Dhatarwal explored the use of ZnO, TiO_2_, and SnO_2_ as nanofillers in PMMA thin films and similarly observed the reduction in the optical band gap of PMMA [117]. In comparison to the optical band gaps reported in their study, the values reported in this work indicate that the material produced exhibits superior optical properties.

### 3.5. Ellipsometry

**Figure 11:**
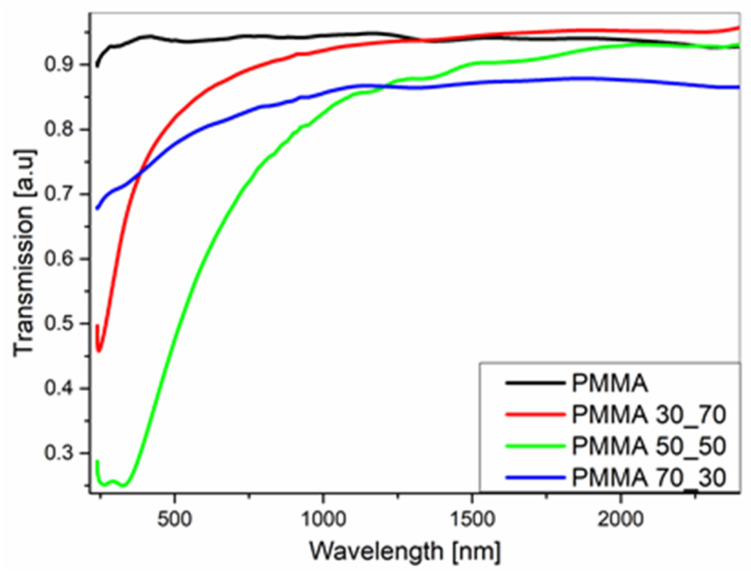
The transmission spectra of PMMA and its composite mats, deposited on quartz substrates.

The transmission spectra of pure PMMA and PMMA composites with different component concentrations, deposited onto quartz, are shown in Figure 11. It has been observed that the addition of dopant has limited the intensity of transmission, especially in the UV range. In the remaining range, the permeability remains at the level of 80–90%.

**Figure 12:**
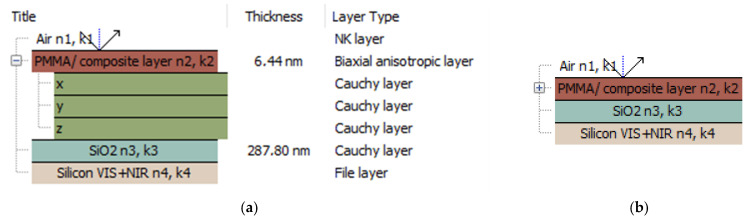
The ellipsometric model applied to PMMA and its composite mats, deposited onto silicon substrates (**a**) and the ellipsometric, not detailed, model applied to PMMA and its composite mats, deposited onto silicon substrates (**b**).

The ellipsometric technique measures the reflectance complex ratio:

(2) $$\rho =e^{i∆}tan\psi ,$$

where the ellipsometric angles—ψ and ∆—are the change in polarisation amplitude, and the phase difference is between the *p* and *s* components of the electric field vector, respectively. The reflectance is the function depending on the complexed dielectric functions of an individual optical setup and has to be determined theoretically. The optical setup, in this case, consists of three layers and is presented in Figure 12. The PMMA and its composite layers were fitted with a biaxial anisotropic model [118], where the refractive indices differ for each axis of the xyz coordinate system.

**Figure 13:**
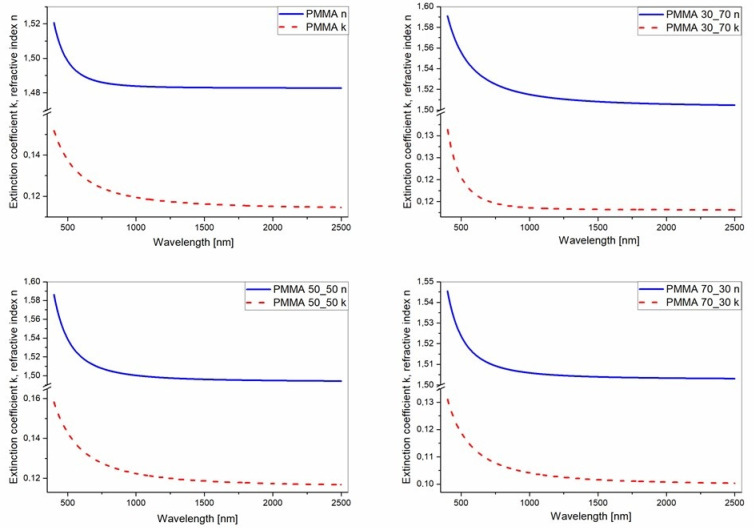
The refractive indices and extinction coefficient dispersions determined for the produced mats.

Individual parameters for xyz directions were fitted using Cauchy layers in all the wavelength ranges. The Cauchy relation parametrises the dependence of the refractive index *n* and the extinction (absorption) coefficient *k* on the wavelength λ. This relation is described by the following equations [34,119,120]:

$$n\left(\lambda \right)=n_{0}+C_{0}\frac{n_{1}}{\lambda^{2}}+C_{1}\frac{n_{2}}{\lambda^{4}},$$

(3) $$k\left(\lambda \right)=k_{0}+C_{0}\frac{k_{1}}{\lambda^{2}}+C_{1}\frac{k_{2}}{\lambda^{4}},$$

where $C_{0}$ and $C_{1}$ are the numerical constants, and *n* and *k* are the coefficients mentioned above. Refractive indices and extinction coefficients, determined for PMMA and its composite films, are shown in Figure 13.

**Figure 14:**
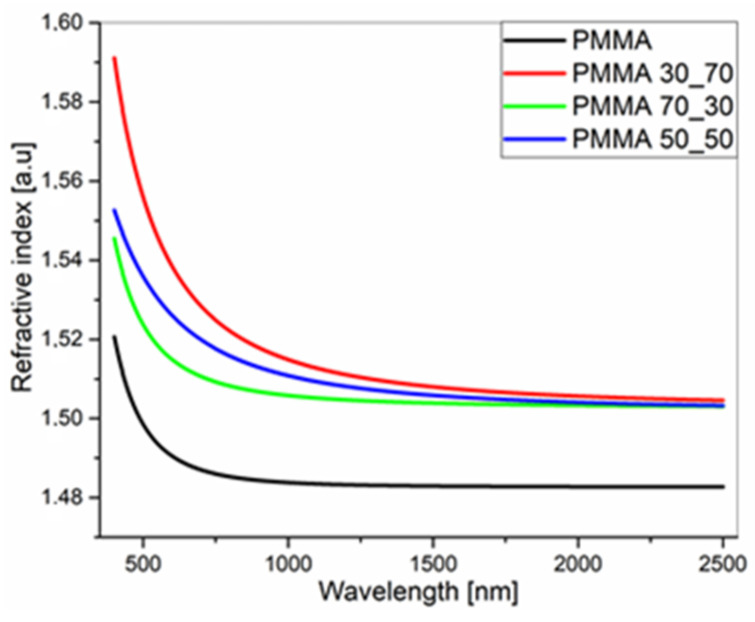
Refractive indices of PMMA and its composite films deposited on silicon substrates.

**Figure 15:**
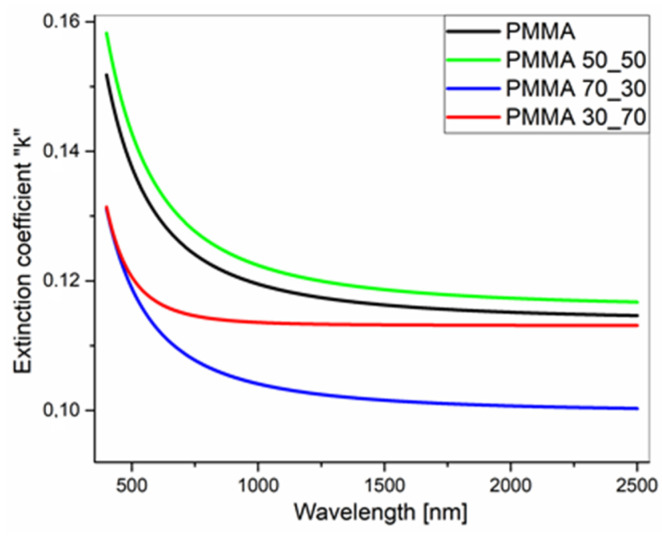
Extinction coefficients of PMMA and its composite films deposited on silicon substrates.

The optical refractive indices, determined for individual xyz directions and for biaxial layers, are presented in Table 2. The dispersions of *n* and *k* for PMMA and its composites are compared to each other in Figure 14 and Figure 15.

**Figure 16:**
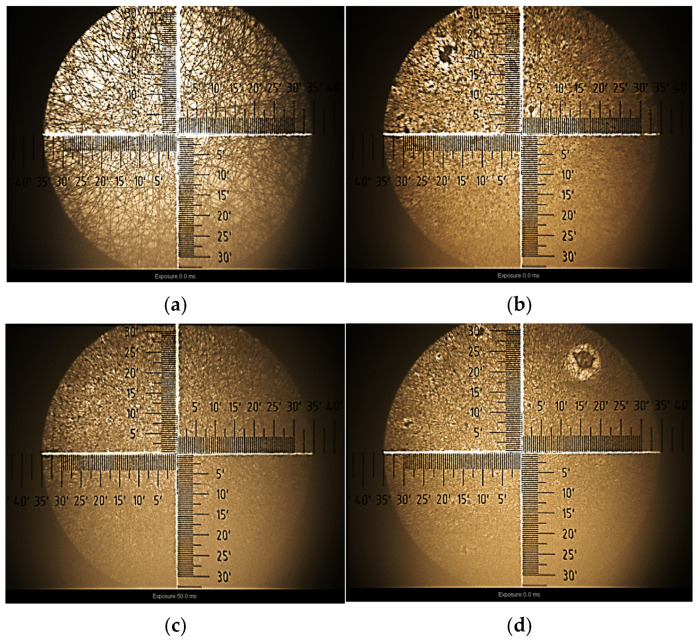
The surface of PMMA film (**a**) and its composites PMMA 30_70 (**b**), PMMA 50_50 (**c**) and PMMA 70_30 (**d**) deposited on silicon substrates.

It is easy to see that the refractive index of the pure PMMA layer is equal to the standard value 1.49, which is well known from the literature [121]. The n coefficients of PMMA composites have slightly higher values. The obtained results, indicating the anisotropy of the refractive indices of the sample, are fully consistent with the pictures of the surface morphology (Figure 16a–d), taken on an optical microscope with a built-in ellipsometer.

## 4. Conclusions

This paper describes a method of making novel PMMA/montmorillonite/Fe_3_O_4_ nanofibrous mats using the electrospinning solution technique. The applied method allows the production of polymer fibre PMMA and composite fibre PMMA/montmorillonite/Fe_3_O_4_ with different concentrations of particles as fillers. The FTIR spectra of the manufactured samples confirmed the chemistry of the fibrous mats by the presence of prominent functional groups. Analysis of the micrograph of the fibrous PMMA and composite mats revealed that the obtained fibres were in the nano regime; consequently, the addition of the nanophase led to the formation of fibres with increased diameters. This was a result of the presence of the nanophase in the spinning solution, which enhanced its viscosity by increasing the solid concentration in the solution. This resulted in fewer beads in comparison to the pristine PMMA fibres. The investigation of the optical properties and band gap of the produced nanofibrous mats revealed that the composite mats had significantly improved absorption of electromagnetic radiation in the ultraviolet and visible light regions compared to the pristine PMMA. The optical band gap calculated for the composite mats was in the 2.5–3.9 eV region, making them suitable for photocatalytic applications. Based on the obtained optical results, we can conclude that the addition of a dopant limited the intensity of transmission, especially in the UV range. Analysis of individual refractive indices showed that pure PMMA had the lowest value of n. In the case of the refractive indices determined for xyz directions at λ = 2500 nm, there is an obvious anisotropic internal morphology of the prepared samples, which is fully consistent with the morphology images obtained using an optical microscope. The results obtained were consistent with the current literature, particularly the values for surface roughness, which aligned with results for materials with an active surface area suitable for interactions with aqueous and gaseous species. This implies that the material produced is suitable for photocatalytic membranes or optoelectronic applications for chemical sensors and protective UV coatings.

## Figures and Tables

**Table 1:** Parameters from AFM analysis.

Sample	Minimum (μm)	Maximum (μm)	Arithmetic Roughness (Ra) (μm)	Root Mean Square Roughness (Rq) (μm)
A	−1.209	1.227	0.211	0.267
B	−0.692	1.860	0.354	0.433
C	−2.278	1.362	0.494	0.611
D	−2.004	2.343	0.462	0.559

**Table 2:** The values of refractive indices determined for xyz directions and for individual biaxial anisotropic layers (for the wavelength λ=2500 nm).

Sample	$n_{x}$	$n_{y}$	$n_{z}$	n (For Biaxial Layer)
PMMA	1.51	1.49	1.47	1.49
PMMA 50_50	1.57	1.50	1.49	1.52
PMMA 70_30	1.52	1.55	1.50	1.53
PMMA 30_70	1.47	1.55	1.49	1.50

## Data Availability

Data is available upon request.

## Supplementary Materials

The following supporting information can be downloaded at https://www.mdpi.com/article/10.3390/polym17030384/s1, Figure S1: Figure of the band plot.
