# Supplementary material for: Tuning the Optical Properties of Electrospun Poly(methyl methacrylate) Nanofibres via Montmorillonite and Magnetite Ratios
Source: Polymers (Basel). 2025 Jan 31;17(3):384. doi: 10.3390/polym17030384 (PMC11820435; doi:10.3390/polym17030384)
Supplement: Supplementary file 1 [file polymers-17-00384-s001.zip › polymers-3398857-supplementary.pdf]

Tuning The Optical Properties of Electrospun PMMA Nanofibers via Montmorillonite and Magnetite Ratios

Yao Mawuena Tsekpo[1*], Weronika Smok[1], Krzysztof Matus[2], Barbara Hajduk[3], Adrian Radoń[4], Paweł Jarka[1] and Tomasz Tanski[1]

[1] Department Engineering Materials and Biomaterials, Faculty of Mechanical Engineering, Silesian University of Technology, Konarskiego 18A, 44-100, Gliwice, Poland

[2] Materials Testing Laboratory, Faculty of Mechanical Engineering, Silesian University of Technology, Konarskiego 18A, 44-100, Gliwice, Poland

[3] Centre of Polymer and Carbon Materials, Polish Academy of Sciences, 34 Marie Curie-Skłodowska Str., 41-819 Zabrze, Poland

[4] Łukasiewicz Research Network – Institute of Non-Ferrous Metals, Sowińskiego 5 St., 44-100 Gliwice, Poland

*Corresponding author's email: yao.tsekpo@polsl.pl

1. Figure of the band plot

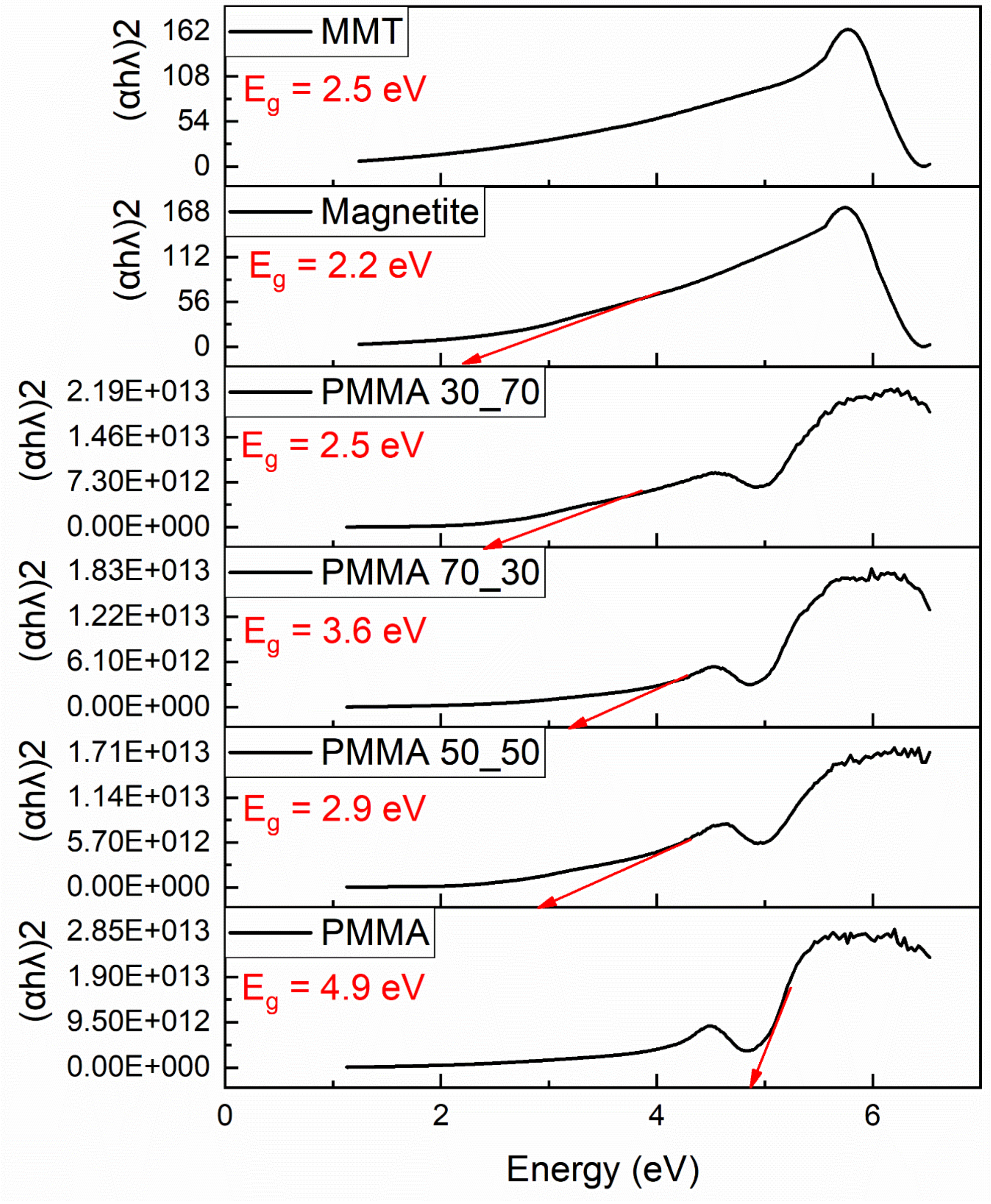

MMT
E_g = 2.5 eV
Magnetite
E_g = 2.2 eV
PMMA 30_70
E_g = 2.5 eV
PMMA 70_30
E_g = 3.6 eV
PMMA 50_50
E_g = 2.9 eV
PMMA
E_g = 4.9 eV
(αhλ)2
162
108
54
0
168
112
56
2.19E+013
1.46E+013
7.30E+012
0.00E+000
1.83E+013
1.22E+013
6.10E+012
1.71E+013
1.14E+013
5.70E+012
2.85E+013
1.90E+013
9.50E+012
0
2
4
6
Energy (eV)
